# Patients’ Non-Medical Characteristics Contribute to Collective Medical Decision-Making at Multidisciplinary Oncological Team Meetings

**DOI:** 10.1371/journal.pone.0154969

**Published:** 2016-05-11

**Authors:** Léa Restivo, Thémis Apostolidis, Anne-Déborah Bouhnik, Sylvain Garciaz, Thérèse Aurran, Claire Julian-Reynier

**Affiliations:** 1 Aix Marseille Université, Laboratoire de Psychologie Sociale EA 849, Aix en Provence, France; 2 INSERM, UMR912 (SESSTIM), Marseille, France; 3 Aix Marseille Université, UMR_S912, IRD, Marseille, France; 4 Institut Paoli-Calmettes, Marseille, France; The University of Hong Kong, HONG KONG

## Abstract

**Background:**

The contribution of patients’ non-medical characteristics to individual physicians’ decision-making has attracted considerable attention, but little information is available on this topic in the context of collective decision-making. Medical decision-making at cancer centres is currently carried out using a collective approach, at MultiDisciplinary Team (MDT) meetings. The aim of this study was to determine how patients’ non-medical characteristics are presented at MDT meetings and how this information may affect the team’s final medical decisions.

**Design:**

Observations were conducted at a French Cancer Centre during MDT meetings at which non-standard cases involving some uncertainty were discussed from March to May 2014. Physicians’ verbal statements and predefined contextual parameters were collected with a non-participant observational approach. Non numerical data collected in the form of open notes were then coded for quantitative analysis. Univariate and multivariate statistical analyses were performed.

**Results:**

In the final sample of patients’ records included and discussed (N = 290), non-medical characteristics were mentioned in 32.8% (n = 95) of the cases. These characteristics corresponded to demographics in 22.8% (n = 66) of the cases, psychological data in 11.7% (n = 34), and relational data in 6.2% (n = 18). The patient’s age and his/her “likeability” were the most frequently mentioned characteristics. In 17.9% of the cases discussed, the final decision was deferred: this outcome was positively associated with the patients’ non-medical characteristics and with uncertainty about the outcome of the therapeutic options available.

**Limitations:**

The design of the study made it difficult to draw definite cause-and-effect conclusions.

**Conclusion:**

The Social Representations approach suggests that patients’ non-medical characteristics constitute a kind of tacit professional knowledge that may be frequently mobilised in physicians’ everyday professional practice. The links observed between patients’ attributes and the medical decisions made at these meetings show that these attributes should be taken into account in order to understand how medical decisions are reached in difficult situations of this kind.

## Introduction

The image of a neutral physician or health care provider taking medical decisions on the sole basis of patients’ medical characteristics has long been challenged in the field of social science [1]. Many studies, mostly in the field of painful disorders, have provided evidence that non-medical factors are usually involved in medical professionals’ everyday practice [2, 3, 4]. Among these non-medical aspects, patients’ social characteristics such as their age, gender and social class have been found to account for the variability of physicians’ decisions [5, 6]. These studies have clearly shown that medical decision-making is rooted in social context. “Non-medical” characteristics have been defined as those which have no clinical or other medical relevance [1]. In the latter review [1], the authors specify, for example, that patients’ gender is a non-medical characteristic when it serves as a marker of their social role and cannot be regarded as being relevant in any way to their disease. The influence of non-medical characteristics has been studied in the context of individual physicians’ decision-making. However, a collective approach to medical decision-making has been adopted as the result of recent changes in medical practice [7, 8]. For the last thirty years, medical decision-making has been framed in terms of the Evidence Based Medicine approach [9], whereby medical decision-making is based on randomised clinical trials and subsequent studies on the lowest levels of evidence. This has been the principle underlying most of the clinical practice guidelines drawn up to assist physicians with their decision-making and standardise patients' management [10]. When scientific data are lacking and medical decision-making cannot be evidence-based, decision-making can be based on either professional guidelines of other kinds or physicians’ own clinical experience of specific cases. This is what happens when dealing with advanced cases of cancer, for example [11]. To handle the complexity of these situations, collective procedures for the management of patients’ care have been introduced. Multidisciplinary Team (MDT) meetings provide a useful means of collective management. These meetings are attended by various hospital specialists, who discuss which therapeutic strategy should be adopted to deal with individual patients. As far as we know, no previous studies have focused so far on the contribution of patient’s non-medical characteristics to collective medical decision-making.

Social Representations Theory, which focuses on the social anchoring of human behaviour and the underlying mental processes [12], provides a useful frame for improving our understanding of how these characteristics contribute to medical decision-making [13]. Studies based on this approach have shown, for example, how medical practices have been shaped by moral and social values [13]. By taking physicians’ judgments and perceptions of patients’ characteristics to be a kind of knowledge, the Social Representations approach invites us to consider the social and practical uses of this knowledge in the contexts in which it is mobilised [13]. These forms of knowledge rooted in affective, social and cultural ground are socially constructed and shared during social interactions, and reflect normative social settings [14]. Like other natural situations in which communication takes place between physicians, MDT meetings constitute an ideal context for studying the use of patients’ non-medical characteristics, which were regarded here as social forms of knowledge.

In the field of oncology, which is a leader in the use of innovative approaches to patients’ care, procedures of this kind have been introduced in many Western countries’ health care systems [8]. In France, all new cases of cancer are registered in the records of MDT meetings, but only complex non-standard cases have to be discussed at these meetings [15].

To compensate for the lack of studies on the influence of patient’s non-medical characteristics on collective decision-making, it was proposed to examine how these characteristics were presented at Multidisciplinary Team meetings in the field of oncology, to determine how frequently these characteristics were mentioned, and to determine how this information was associated with the outcome of the collective medical decision-making process.

In line with the Social Representations approach, which takes patient’s non-medical characteristics to be a kind of knowledge which gives people the feeling of mastering the situation, we focused here on Multidisciplinary Team (MDT) meetings dealing specifically with non-standard case records involving a high level of uncertainty, at which non-medical information was likely to crop up fairly frequently. It was therefore proposed to study these issues by performing quantitative assessments on data collected at MDT meetings at a French Comprehensive Cancer Centre. Conducting field observations during MDT meetings provided a suitable opportunity for systematically noting spontaneous references to patients’ non-medical characteristics in the context of collective decision-making.

## Methods, Choice of Setting, Data Collection, Statistical Analysis

### 2.1. Methods

Data including physicians’ verbal statements and a pre-defined set of characteristics describing the context of the MDT meeting and the cases under discussion were collected using a non-participant observational approach during MDT meetings conducted at a single comprehensive cancer centre in France. Physicians’ verbatim statements were then coded systematically before being analysed using quantitative methods.

The study protocol, including the consent procedure, was approved by the Institutional Review Board of the French National Health and Medical Research National Institute (INSERM) (approval number 14–144). Application was made by mail to the Institution’s legal and managerial departments, which sent their approval by return mail. The four MDT leaders were asked whether they agreed to having their meetings observed for the purpose of this study. They all gave their written consent by return mail. At the beginning of the first MDT meeting, the main researcher, who acted as the observer, and the aims of the study were presented to all the participants, who were asked to give their verbal consent to the meeting being observed. Written consent would have made the issue of confidentiality more complex to handle. All the participants agreed verbally to the researcher being present during their meetings, and this fact was recorded in the minutes of the first meeting.

### 2.2. Choice of setting

#### 2.2.1 Institution selected

The institution at which this study was conducted was the place where the members of the social science research team who wrote this paper worked. The fact that we had been working for a long time with clinicians belonging to this institution facilitated our access to their meetings. This institution is one of the eight French regional comprehensive cancer centres (labelled "SIRIC" centres by the French National Cancer Institute) which are involved in both research and clinical practice. Standardised cancer patient management procedures such as MDT meetings have been adopted at this institution, which handles many patients with many different kinds of cancer and therefore gives us an opportunity of collecting large bodies of data of various kinds. The procedures adopted here correspond to what occurs at the other comprehensive cancer centres and French university teaching hospitals’ oncology wards. The practice of discussing "newcomer cases" at separate MDT meetings from "relapses and non-standard cases" at this institution facilitated the collection of data of interest in this study.

#### 2.2.2. The type of MDT meeting selected

The type of MDT meeting at which this study was conducted was that where the treatment for the cases reviewed was the least likely to be”evidence-based” or recommended in the guidelines available. Since it was assumed that patients’ non-medical characteristics were most likely to be mentioned in situations involving uncertainty about the best therapeutic option, MDT meetings of four kinds dealing with advanced cancer cases, relapsing cases and cases with a poor prognosis were selected. The first MDT meeting was about “Metastatic Breast Cancer”, the second one was about “Myeloid Haematology”, the third one was about “Lymphoid Haematology” and the last one was about “Gastro-intestinal-Pancreatic cancer”.

#### 2.2.3. Criteria adopted to select the cases included in the study

The cases analized here were those where the following information was available: the reason for presenting the case, the medical options discussed and the final decision.

The cases excluded were those where guidelines could be applied without any need to discuss which treatment should be adopted, and secondly, those removed from the agenda of the MDT session either because they would have to be discussed at a future session since key medical information was missing or because they had to be referred to a MDT meeting of a different kind.

### 2.3. Data collection

#### 2.3.1. Dataset characteristics

During the observation period, 48 MDT meetings on cases of the four types listed above took place at the institution, corresponding to 532 complete patients’ case records. The meetings were held every week in the morning (lymphoid haematology), evening (metastatic breast and digestive cancer) and at lunchtime (myeloid haematology). These consecutive meetings were systematically attended by the main researcher (LR) from March to May 2014 at the institution in question. To reduce the risk of Hawthorn effect, the participants at MDT meetings were informed that the main aim of the study was: "to study medical decision-making in oncology", without giving any details which might have influenced what the participants said. In order to minimize this risk, the observer attempted to behave as discreetly as possible and to avoid interacting with the participants during the meetings.

Two observation grids, one for noting the characteristics of the MDT meetings and the other for noting the case-record characteristics, were drawn up, based on the literature [16, 17]; they were gradually adapted during a previous pilot observation period which focused on six MDT meetings and at workshops in which a stepwise process of discussion was used between the researchers involved (LR, CJR and TA). The purpose of these grids was to focus our observations of the meetings on a set of specific variables which seemed to be relevant to the aims of our study, and were used to ensure that the outcome variables would be collected systematically.

Data collection at MDT meetings:The observation grid was initially designed in the form of a set of themes, closed questions and pre-coded items corresponding to the duration of the meeting, the number of doctors present, the number of doctors discussing the cases, the number of cases discussed, and the number of cases removed from the agenda.

Case data collection: The collection of case data was based on the use of the predefined open questions composing the observation grid, about the patient’s sex and age, the reason for presenting the case, the options involved, the final decision, the duration of the case discussion, the presence or absence of the doctor in charge of the patient, the number of doctors discussing the case, and the preferences expressed by the patient about the treatment. The observers took notes on a separate pre-formatted sheet of paper during each case discussion, without any *a priori* assumptions about the content of the variables. The task of recording every mention of patients’ non-medical characteristics, the main outcome of the study, was facilitated by the fact that these characteristics could be mentioned only by physicians who were in charge of the patient and were present at the meeting (just one physician most of the time). The task of collecting the other information of interest was also simplified by the standardised presentation of the clinical cases and the fact that the observer had become familiar during the pilot study with cancer patients’ medical characteristics.

#### 2.3.2. Coding process

MDT data: Data collected on the observation grid did not need to be transformed for the statistical analysis. They were fed directly into the computer for processing.

Case data: from physicians’ verbatim statements to quantitative analysis: In order to obtain a quantitative assessment of the physicians’ verbatim statements, the content of the variables included in the observation grid was coded by the authors with a view to drawing up a closed thematic grid for the statistical analysis of the results of the observations. Six workshops were attended by the researchers (LR, CJR, ADB and TA) in order to define and harmonise the categories which emerged from the open notes, at which 25 of the cases addressed at the 4 MDT meetings were systematically presented (by LR). Whenever any new categories emerged, they were systematically added to the coding sections. To check the validity of the coding, a randomly selected sample of cases (n = 36) was then taken and coded independently by three coders (LR, SG and VA), two of whom were physicians familiar with what occurs at MDT meetings. Each coder working independently used the final closed thematic grid to code the cases presented orally by LR, based on the observation notes. Any discrepant records were reviewed by the coders at a special workshop, and rules for systematising the coding process were then drawn up. These rules were applied whenever these particular situations emerged in the cases observed. With the most complex cases, special workshops attended by the three main researchers (LR, ADB and CJR) were held in order to obtain a consensual collective coding system.

Dependent variables: Patients’ non-medical characteristics were categorised based on the coding of the patients’ psychological, relational and socio-demographic characteristics mentioned at the collective MDT discussions. The coding procedure used is presented in Table 1.

**Table 1:** A case-vignette illustrating the coding process used in this study on patients’ non-medical characteristics.

Non numerical data: summary of the case vignette and extracts from observers’ notes	Analysis of the case by the research team	Quantitative Coding of the patient’s non-medical characteristics
Mr X, a 56-year old colon cancer patient with liver metastasis.	This case discussion, focused mostly on the patient’s unusually young age (he was only 56 whereas this condition usually occurs fifteen years later at the age of about 70–75) (“young man” was mentioned 3 times). Several of the practitioners present stated that a surgical intervention was relevant and possible; others said that the patient had already undergone enough treatment and that a surgical intervention would be “too much”. One doctor assumed that some practitioners willing to prescribe surgery thought this should be attempted because of the patient’s age although this conclusion was not found to be relevant using an evidence based approach.	Category: Socio-demographics.
**Reason for discussion at the MDT meeting:** To select the best therapeutic alternative from a range of options available.		Sub-category: Age.
**3 Options discussed:** Giving the treatment a break or performing surgery or chemotherapy.		
**Context of the discussion:** One of the physicians present clearly stated that he was in favour of giving the treatment a break and against surgery. About the surgical option discussed at the MDT meeting, he said: “We are being too aggressive because of his age. The prognosis is the same at 56 as at 75.”	***Conclusion of the research team*:** in this case, age was not used as a medical criterion but as a non-medical characteristic. Age was regarded here as a social value in the discussion which took place at the MDT meeting.	
Mr X, a 65-year old haematological cancer patient.		Category: Psychological characteristics.
**Reason for discussion at the MDT meeting:** To select the best therapeutic alternative from a range of options available.		Sub-category: a fighter, active.
**Options discussed:** treatment or no treatment.		
**Final decision at the MDT meeting**: left to the patient to decide, depending on his own personal preferences.	***Conclusion of the research team*:** in this case, the active profile of the patient emerged as a non-medical characteristic which intervened in the discussion. In the decision-making process, this characteristic was used to balance the patient’s clinical condition.	
**Statement by one of the physicians discussing the case and the patient:** “A man who is doing well, who has lots of projects but is still disabled […] He is disabled but he is a very active person.”		
Ms X, a 37-year old patient with face nodules (tumoral mycosis).		Category: Relational characteristics.
**Reason for discussion at the MDT meeting:** To select the best therapeutic alternative from a range of options available.		Sub-category: Attachment.
**Options discussed:** Chemotherapy or “wait and see” and subsequent re-assessment.		
**Final decision adopted at the MDT meeting:** subsequent re-assessment.		
**Citation of the physician in charge of the patient:** “I felt I couldn’t’ jut do nothing for her, so I put her on Purinetol (…) In addition, she is very appealing this girl, that is, her social life is miserable but she is very appealing.”	***Conclusion of the research team*:** in this case, the attachment of the healthcare providers to the patient turned out to be a non-medical characteristic which intervened in the discussion.	

Deferring the final conclusions of the MDT meetings—It was sometimes decided at the MDT meetings to defer the final decision about the choice of treatment. The final decision could be postponed to a subsequent MDT meeting (but not because of missing medical information, since these cases were excluded from the analysis at the start). It could also be left to the patients to decide about the treatment proposed, depending on their own preferences. And lastly, the final decision among the various options presented at the MDT meeting could be left to the physician in charge of the patient.

Independent variables: Type of MDT meetings: Metastatic Breast Cancer, Myeloid Haematology, Lymphoid Haematology, Gastro-Intestinal/Pancreatic Cancer.

The other characteristics of MDT meetings were also noted (duration of the meeting, number of doctors present, number of doctors discussing the cases, number of cases discussed, number of cases cancelled from the agenda).

Sociodemographic characteristics (age, gender) and several characteristics of the decisional context were noted in connection with every case presented (duration of the discussion, presence or absence of the doctor in charge of the patient, number of doctors discussing the case, whether preferences expressed by the patient about the treatment were mentioned).

On each patient’s report sheet, the process of medical decision making was systematically noted:

- Five main reasons for presenting the case at the MDT meetings were coded: 1) to select the best therapeutic alternative among a range of options available 2) a standard decision needing to be discussed 3) no treatment options available any longer 4) further diagnostic investigations required 5) inclusion in clinical trials.
- Seven management options discussed at the MDT meetings were coded separately: 1) the availability of various therapeutic protocols corresponding to the same category of treatment, such as two different chemotherapy protocols. 2) invasive vs non-invasive interventions 3) standard treatment vs clinical trials 4) treatment or no treatment 5) treatment or further diagnostic investigations 6) different treatments involving similar risks 7) a consensually accepted case management option.
- Uncertainty about the outcomes of the medical options discussed was coded as present or absent.

### 2.4. Statistical analysis

Chi-square tests and Student’s t-tests were used to compare the characteristics of the MDT meetings and case records among the four different types of MDT meetings. Univariate analyses were then performed in order to identify the factors associated with our two variables: patients’ non-medical characteristics and deferring the final conclusion reached at the MDT meeting. A multivariate analysis was performed on each variable in order to identify independently associated factors, using logistic models. All the factors found to be associated with a variable with a p-value <.20 in the univariate analysis were taken to be eligible for the multivariate model. Only factors still significantly associated with a p <.05 were kept in the final model. Statistical analyses were performed using PASW Statistics 18 v18.0.3 software.

## Results

### 3.1. Description of the MDT meetings

Forty-eight consecutive MDT meetings were observed and subsequently analysed. The four types of MDT meetings differed significantly in terms of their overall duration and all the other characteristics such as the number of doctors attending the meeting, those participating in the discussions, and the number of cases discussed. However, the number of cases removed from the agenda was similar at all four types of meetings observed. Details about each type of meeting are given in Table 2.

**Table 2:** MDT meeting characteristics.

Characteristics of MDT meetings (N = 48)	Metastatic breast cancer (n = 14)	Myeloid Haematology (n = 9)	Lymphoid Haematology (n = 12)	Liver/Pancreas (n = 13)	Total (N = 48)	P-value^a^
	Mean (SD) [Min-Max]	Mean (SD) [Min-Max]	Mean (SD) [Min-Max]	Mean (SD) [Min-Max]	Mean (SD) [Min-Max]	
**Duration (hours and minutes)** (N = 48)	1:24 (0:32) [0:30–2:24]	0:47 (0:23) [0:08–1: 21]	1:25 (0:15) [0:55–1: 52]	1:52 (0:36)[1: 00–2: 48]	1:25 (0:35)[0:08–2:48]	<0.001
**Number of doctors present** (N = 48)	6 (2.4) [2–10]	6 (2.1) [2–8]	10 (2.1) [6–13]	10 (2.2) [6–14]	8 (3.0) [2–14]	<0.001
**Number of doctors discussing cases** (N = 47)	4 (1.0) [2–5]	4 (1.6) [2–6]	6 (1.6) [3–8]	6 (1.6) [3–9]	5 (1.8) [2–9]	<0.001
**Number of cases discussed** (N = 48)	14 (4.4) [7–23]	8 (3.5) [2–13]	16 (3.3) [11–21]	21 (7.4) [12–36]	15 (6.5) [2–36]	<0.001
**Number of cases removed from the agenda** (N = 48)	3 (2.0) [0–6]	3 (2.0) [0–6]	2 (1.9) [0–7]	4 (3.6) [0–14]	3 (2.5) [0–14]	0.610

^a^ Using Anovas.

### 3.2. Description of the cases discussed

**Fig 1:**
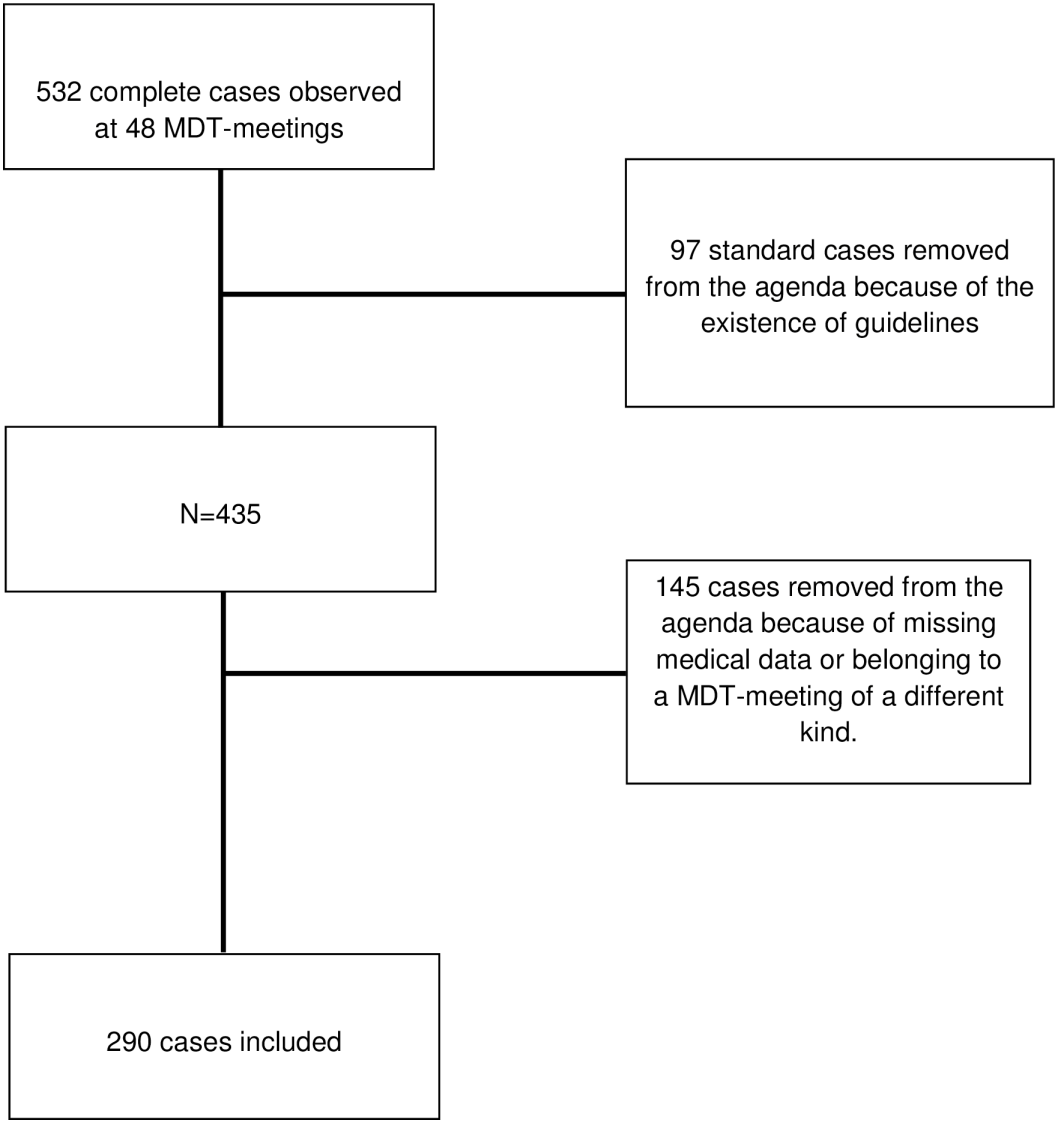
Strategy of case-records selection for the analysis.

Among the 532 complete case-records scheduled for discussion at the MDT meetings, 290 patients’ cases were included in the analysis (Fig 1). The cases excluded were 242 cases that did not meet the inclusion criteria either because no discussion was required about their medical management or because they were removed from the agenda of the MDT meeting (cases that had to be discussed at a future session because key medical information was missing or were referred to a MDT meeting of a different kind) (Fig 1). The patients’ mean age was 61.1 (SD = 14.3) and their gender is given in Table 3. The average duration of each case discussion was 7mn (SD = 4.0).

**Table 3:** Characteristics of the cases: Context of the decision-making process.

Characteristics of the cases presented at the four MDT meetings (N = 290)	Total (N = 290)
	n (%)
**Non-medical characteristics spontaneously mentioned in the case discussion (N = 290):**	
Yes	95(32.8)
No	195(67.2)
**Deferring the final conclusion at the Multidisciplinary Team Meeting (N = 290):**	
Yes	52(17.9)
No	238(82.1)
**Sex** (N = 282)	
Men	118 (41.8)
Women	164 (58.2)
**Presence of the physician in charge of the patient (N = 290):**	
Yes	178(61.4)
No	112(38.6)
**Practitioners taking part in discussions (N = 210)**	
n ≤2	91(43.3)
n >2	119(56.7)
**Patient’s preferences for treatment mentioned (N = 290):**	
Yes	30 (10.3)
No	260 (89.7)
**Options discussed at the MDT meeting (N = 290):**	
Different therapeutic protocols possible in the same category of treatment	179(61.7)
Invasive *vs* Non invasive intervention	15(5.2)
Standard treatment *vs* clinical trials	22(7.6)
Treatment or no treatment	23(7.9)
Treatment or further diagnostic investigations	16(5.5)
Different therapies involving equivalent risks	7(2.4)
A single consensually accepted option available	28(9.7)
**Uncertainty about the outcomes of the various options (N = 290):**	
Yes	215 (74.1)
No	75 (25.9)

### 3.3. Description of the collective decision-making process

#### 3.3.1. The decision-making context

The physician in charge of the patient was present in 61.4% of the case-by-case discussions. More than two physicians participated in the discussions in 56.7% of the cases. Patients’ specific preferences were mentioned in 10.3% of the cases (Table 3).

#### 3.3.2. Reason for presenting patients’ records at the MDT meetings

In 82.8% of the cases (n = 240), the reason for presenting the patients’ records at the MDT meetings was to select the best therapeutic alternative from the range of options available; the other reasons were that a standard decision was available but needed to be discussed (11%; n = 32), no treatment options were available any longer (treatment dead end) (3.1%, n = 9), further diagnostic investigations were required (1.7%, n = 5), and the possibility of inclusion in clinical trials (1.4%, n = 4).

#### 3.3.3. Medical options discussed at the MDT meetings

The most frequently discussed option in 61.7% of the cases was that various therapeutic protocols corresponding to the same category of treatment were available. The second most frequent situation, which occurred in less than 10% of the cases discussed, was that one option was consensually accepted by all those attending the meeting. Five other options were presented less frequently (Table 3).

#### 3.3.4. Uncertainty about the options presented

Uncertainty about the outcomes of medical options was present in 74.1% of the cases.

#### 3.3.5. Deferring the final conclusion at the MDT meetings

The final decision about the choice of treatment was deferred at the MDT meetings in 17.9% (n = 52) of the cases discussed. In 8.6% of the cases (n = 25), the final decision was postponed to a subsequent MDT meeting, in 5.5% (n = 16) it was left to the patients to decide, depending on their own personal preferences. And lastly, in 3.8% of the cases discussed (n = 11), the final choice among the options presented at the MDT meeting was left to the physician in charge of the patient.

### 3.4. Mention made of patients’ non-medical characteristics and related factors

Patients’ non-medical characteristics were mentioned during the discussions in 32.8% of the cases. In 22.8% (n = 66) cases, they were sociodemographic characteristics, in 11.7% (n = 34) cases, they were psychological ones, and in 6.2%(n = 18) cases, they were relational ones. Details about the type and frequency of occurrence of these characteristics are given in Table 4. Age and occupation were the most frequently mentioned socio-demographic patients’ characteristics. The psychological characteristics most frequently mentioned by physicians during the discussions focused on patients’ likeability (“nice” or “annoying”). The relational characteristics mentioned by physicians related to patients’ family or the physicians’ relationships with their patients.

**Table 4:** Distribution of patients’ non-medical characteristics mentioned at MDT meetings (N = 95 cases).

Non-medical characteristics (N = 132)	Type of contents	Frequencies [n]
**Socio-demographic (n = 74)**		
	Age	42
	Occupation	13
	Place of abode	6
	Origin, nationality, ethnic specificities	5
	Patients having physicians in their family	4
	Religious beliefs	2
	Marital Status	2
**Psychological (n = 39)**		
	Nice patient	10
	Annoying patient	6
	Strange	5
	Psychologically weak	5
	Cognitive ability (comprehension)	4
	Outsider	2
	Fighter, active	2
	Physically weak	2
	Patients with bad relationships with their relatives	2
	Patient who never complains	1
**Relational (n = 19)**		
	Bothersome family	5
	Attachment	5
	Patient making strong demands	3
	Beautiful appearance	3
	Compliance	1
	Argumentative	1
	Nationality, origins^a^	1

^a^ Patients’ nationality and origins were categorised as relational characteristics when they were mentioned in connection with the physician own nationality or origin.

These characteristics were more frequently mentioned at the Metastatic Breast Cancer MDT meeting (adjusted OR 2.21 95% Confidence Interval 1.10–4.44) when the patients’ preferences were expressed (adjOR: 2.94 95% CI: 1.25–6.89) and when the physician in charge of the patient was present (adjOR: 2.06 95% CI: 1.14–3.73). The rate of occurrence of patients’ non-medical characteristics increased significantly (p <.05) with the time spent discussing the case (Table 5).

**Table 5:** Factors associated with the mention of patients’ non-medical characteristics: univariate and multivariate comparisons (logistic regression).

	Non-medical characteristics mentioned during the MDT discussions (N = 290)						
	Yes (32.8%)	No (67.2%)			Multivariate adjustment (N = 252)		
	n(%)	n(%)	P-value	Crude odds ratios	Adjusted odds ratios	95% CI	P-value
**Type of MDT meeting type (N = 290)**							
Metastatic Breast cancer	37(42.5)	50(57.5)	0.020	1.8	2.2	1.1–4.4	0.025
Other forms of cancer (Myeloid haematology, Lymphoid haematology, Liver/pancreas)	58(28.6)	145(71.4)					
**Patient's preferences expressed (N = 290)**							
Yes	17(56.7)	13(43.3)	0.003	3.1	2.9	1.3–6.9	0.013
No	78(30.0)	182(70.0)					
**Duration of case discussion (N = 283)**							
Less than or equal to 4 minutes	20(22.7)	68(77.3)	0.047				
Between 5 and 7 minutes	39(34.8)	73(65.2)		2.2	2.0	1.0–4.0	0.053
Between 8 and 25 minutes	33(39.8)	50(60.2)		2.2	2.8	1.3–5.9	0.007
**Physician in charge of the patient present at the MDT meeting (N = 290)**							
Yes	67(37.6)	111(62.4)	0.026	1.8	2.1	1.1–3.7	0.017
No	28(25.0)	84(75.0)					

No significant associations (p>.05) were found to exist between patients’ non-medical characteristics being mentioned in the discussion and treatment options being discussed at the MDT meeting, the reason for presenting the case at the MDT meeting or uncertainty about the options presented.

### 3.5. Patients’ non-medical characteristics and other factors associated with deferral of the final decision at the MDT meetings

The characteristics associated with the final decision being deferred at the MDT meetings are presented in Table 6. The final decision was deferred most frequently at Metastatic Breast Cancer MDT meetings than at meetings of other kinds (adjOR 2.54 95% CI 1.31–4.92); but the most significant factor was uncertainty: when uncertainty was expressed about the outcomes of the medical options, the final decision was more frequently deferred (adjOR 4.73 95% CI 1.75–12.83). Patients’ non-medical characteristics were found to be significantly positively associated in the univariate comparisons with deferral of the final decision (p = 0.009) and borderline significant after multivariate adjustment (p = 0.056).

**Table 6:** Factors associated with final decisions made at MDT meetings: Univariate and multivariate comparisons (logistic regression).

	Final decision deferred at the MDT meeting (N = 290)						
	Yes (17.9%)	No (82.1%)			Multivariate analysis (N = 259)		
	n (%)	n (%)	P-value	Crude odds ratios	Adjusted Odds ratio	95% CI	P-value
**Type of MDT meeting (N = 290)**							
Metastatic Breast Cancer	23(26.4)	64(73.6)	0.013	2.2 [1.7–4.0]	2.5	1.3–4.9	0.006
Other types of cancer (MDT types Myeloid haematology, Lymphoid haematology, Liver/pancreas)	29(14.3)	174(85.7)					
**Uncertainty about the outcomes of the options (N = 290)**							
Yes	47(21.9)	168(78.1)	0.003	3.9 [1.5–10.3]	4.7	1.7–12.8	0.002
No	5(6.7)	70(93.3)					
**Non-medical characteristics mentioned in the discussion (N = 290)**							
Yes	25(26.3)	70(73.7)	0.009	2.2 [1.2–4.1]	1.9	1.0–3.5	0.056
No	27(13.8)	168(86.2)					

## Discussion

As far as we know, this is the first observational study on whether patients’ non-medical characteristics are mentioned and how this information may contribute to collective medical decision making in the context of oncological MDT meetings at which non-standard cases are discussed. Patients’ non-medical characteristics were mentioned in one third of the cases discussed by physicians: these characteristics mostly took the form of socio-demographic attributes such as age, but psychological and relational ones were also mentioned. These characteristics featured twice more frequently when the physician in charge of the patient was present during the discussion and three times more frequently when the patient’s treatment preferences were stated. These rates of occurrence increased with the time spent discussing the case. Final decisions were deferred at MDT meetings in less than two cases out of ten, and this outcome occurred more frequently when non-medical characteristics were mentioned, especially at the metastatic breast cancer MDT meeting. The presence of uncertainty in the decision-making process was the main factor associated with deferring the final conclusion at the MDT, as it multiplied this outcome fourfold.

### 4.1. Patients’ non-medical characteristics in the context of cancer MDT meetings

The influence of non-clinical criteria such as sociodemographic information on medical decision-making has been described extensively in the context of individual medical practices. Age, followed by gender, race, and economic status have been the most frequent non-medical characteristics discussed in previous North-American studies [1, 6]. In the present study conducted in a collective medical decision making context, age was found to be by far the most frequently mentioned attribute. The fact that physicians often refer to age in their discussions is in line with the social value of younger lives and the need to fight to save younger patients although the prognosis is not age dependent, as established previously [18].

The influence of non-clinical criteria such as patients’ psychological and relational characteristics in medical decision-making has been investigated recently in the context of individual practices [6]. The latter authors stressed the difficult psychological profiles that health practitioners have to cope with, such as those of violent and aggressive patients, whereas both positive and negative characteristics were mentioned by the hospital practitioners involved in the present study, where the ‘nice’ patients were those most frequently described (Table 4). The patients’ family caregivers have been described as patient’s advocates who intervene in the context of end of life decision-making [6, 19]. As far as we know, no other studies have highlighted the role of the family as one of the characteristics mentioned by the practitioners themselves in the context of their collective decision making processes In the present study, the family was mentioned in the collective discussions either to specify that a member of the patient’s family was a physician or when the patient’s relatives were said to be “annoying”. Physicians might be encouraged to mentioning family characteristics of this kind at these meetings because of the medico-legal implications. Other authors have mentioned [20] that these social, cognitive and psychological aspects need to be documented further and would be worth studying more closely. They constitute characteristics that may explain the differences between physicians’ decisions’ more significantly than demographics.

### 4.2. Deferred decisions and associated factors

Previous studies on the treatment decisions made at MDT cancer-related meetings have focused on the participants’ ability to reach a final decision and the factors involved, such as lack of information and organisational factors such as time or the practitioners’ professional profiles [21]. Many experimental studies have dealt with the issue of choice deferral [22]; in the medical setting, several studies have highlighted the significant effects of the number of alternatives available, which contribute increasingly to decision deferral [23, 24]. In the context of several alternatives with an equivalent level of evidence, whatever the effectiveness or the side-effects of the treatments envisaged, uncertainty about which is the most suitable option is certainly an issue. Since the research setting adopted here was one where evidence based medical guidelines are not frequently available, several medical alternatives were possible for treating most of the cases reviewed at the MDT meetings. The large number of clinical trials conducted in the context of metastatic breast cancer patients’ care may explain why it was observed in this study that deferral of the final decision was significantly associated with MDT meetings of this particular kind. In the case of clinical trials, the final decision has to be taken both by the physician in charge of the patient and by the patient him/herself. Clinical trials are indeed specific interventions involving detailed information about the patients and negotiations with them.

The results of this study confirm that uncertainty contributes importantly to deferring final decisions. Various ways of coping with uncertainty have been identified in the literature [25]. In an observational study of MDT meetings, Castel [7] has described, for example, how MDT meeting processes are perceived by physicians as a means of dealing with their experience of uncertainty. By contrast, uncertainty no longer seems to have been a collective matter in the present study since the final decision was deferred in various ways, such as giving the last word to the physicians in charge of the patients or to the patients themselves. It is possible that deferring final decisions may be a strategy used to cope with the uncertainty involved in situations of this kind, where the central role of the patient/physician relationship seems to be restored.

### 4.3. A socio-representational interpretation of patients’ non-medical characteristics in the context of MDT meetings

The setting in which this study was carried out was selected in keeping with the Social Representations approach adopted. In this theoretical framework, people are assumed to construct their own social reality based on multiple forms of knowledge, including social ones, especially when they are faced with unfamiliar situations [26]. In the present setting, patients’ non-medical characteristics, regarded as a form of social knowledge, could be a means of coping with unfamiliar non-standard situations involving uncertainty. The simple fact that patients’ non-medical characteristics were mobilised spontaneously here in real collective decision-making situations supports the idea that these characteristics are “natural” kinds of knowledge existing in social reality, which are used by the social actors involved. Deferring final decisions was found here to be associated with patients’ non-medical characteristics and with uncertainty. Using a Socio-representational approach to interpret these results led us to wonder not whether patients’ non-medical characteristics are relevant factors worth taking into account in medical decision-making, but what purposes these characteristics may serve. Asking this question brings us to question the context in which these characteristics are mentioned. One must remember that the aim of the present MDT meetings was to discuss cases consisting mainly of patients with advanced and relapsing cancer. Situations of this kind are characterized by the fact that physicians have few clinical guidelines on which to base their decisions, and secondly, by their potentially high emotional content. These cases, which are often discussed after several therapeutic failures, tend to involve long-term relationships between the patients and their physicians and the medical staff, and considerations about physicians’ traditional curative role are no longer relevant. In situations of this kind, deferring decisions could be interpreted as means of enabling physicians to face the lack of scientific arguments on which to base their decisions, as well as mastering the socio-emotional issues involved and keeping them at a distance. Mentioning patients’ non-medical characteristics in these situations makes the patients become socially as well as psychologically situated individuals. In other words, the patients are not viewed only from the biomedical perspective (i.e., in terms of the medical characteristics of their disease alone). Their description acquires something of the socio-affective atmosphere of the clinician/patient relationship, whether it is positive (a “nice patient”) or negative (an “annoying patient”). On similar lines, the patients' non-medical characteristics contributed most importantly to the decisions taken when the two main persons involved in the therapeutic relationship were both present (physically in the case of the physician and symbolically in that of the patient when the latter's preferences were mentioned). The fact that more time was spent discussing those cases in which patients’ non-medical characteristics were mentioned suggests that the specific socio-relational factors involved in these cases should be more carefully taken into account. Generally speaking, this finding supports the idea that the social context should be taken into account in order to understand medical decision-making, since it is the place where people's social insertion [5] and also their social participation occur.

### 4.4. Limitations of the study

Several limitations of our study are worth discussing. First, the reason why these MDT meetings were not tape-recorded was to reduce the possibility of self-censorship by the physicians, and the non-numerical data were therefore directly observed and collected. After an initial feasibility study, it was decided to systematise the data collection procedure by drawing up an observation grid and to triangulate the coding procedures. Secondly, the present MDT meetings were specific to very severe forms of cancer on which fewer evidence based guidelines are available than in most other contexts. This was done on purpose to select contexts with non-standard cases. Our results showing that in one third of the cases, non-medical characteristics were mentioned in the context of collective medical decision-making at cancer MDT meetings cannot be extended to more standard cases of cancer. In the latter context, this proportion is likely to be lower. Since these cancer-related MDT meetings involved specialized hospital physicians, surgeons, oncologists, pathologists and radiologists, our results are likely to be comparable to what occurs at all specialized national and international centres.

Because of the design of the study, it was not possible to infer causal relationships between non-medical characteristics and the medical decision outcomes. However, the observational and quantitative approaches adopted in this study yielded some interesting results and hypotheses that need to be studied more closely in order to understand the role of patients’ non-medical characteristics in medical decision-making. Further studies based on in-depth interviews on physicians’ perceived role in the specific situations observed in this study would be a useful means of reaching a fuller understanding of the logics underlying the use made by hospital practitioners of these patients’ non-medical characteristics in their everyday practice.

### 4.5. Conclusion

The results obtained in the present study show that patients’ sociodemographic, psychological and relational characteristics were mentioned by oncologists in one third of the cases discussed in collective medical decision making settings, and that mentioning these characteristics was significantly associated with deferral of the final decision. Deferral of the final decisions occurred in less than one fifth of these cases and was also found to be associated with the presence of uncertainty in the decision making process. In their clinical practice, oncologists are encouraged to take information about their patients’ psychosocial background into account, including their place of abode and the support provided by their close family circle, to enable them to adapt their practices to the patients’ individual specificities. Understanding how this information contributes to collective medical decision making is therefore an important issue. It is likely to be all the more important in the case of Multidisciplinary Team Meetings dealing with non-standard situations involving uncertainty. It is worth mentioning here that although the collective conclusions reached at the present MDT meetings were transmitted in the form of recommendations to the physicians in charge of the patients, the final medical decisions were left to the doctors and their individual patients.

## Supporting Information

S1 File MDT meetings characteristics data set. (SAV)

S2 File Cases characteristics data set. (SAV)
